# Genetic variants in patients with recurrent pericarditis

**DOI:** 10.2459/JCM.0000000000001669

**Published:** 2024-09-17

**Authors:** Massimo Imazio, Flavio Faletra, Jessica Zucco, Catia Mio, Matteo Carraro, Alberto Maria Gava, Marzia De Biasio, Giuseppe Damante, Valentino Collini

**Affiliations:** a Department of Medicine (DMED), University of Udine; b Cardiology and Cardiothoracic Department; c Institute of Medical Genetics, University Hospital ‘Santa Maria della Misericordia’ (ASUFC), Udine, Italy

**Keywords:** genetics, inflammasome, recurrent pericarditis, whole exome sequencing

## Abstract

**Aims:**

Presence of family cases and multiple recurrences of pericarditis suggest the existence of a possible genetic background in at least 10% of cases. The aim of the present study is to describe the genetic landscape of a cohort of patients with multiple recurrences (at least two recurrences).

**Methods:**

Retrospective cohort study of consecutive adult patients referred for at least two episodes of recurrences in a tertiary referral centre. Genetic testing was performed by whole exome sequencing (WES).

**Results:**

Our cohort included 108 consecutive patients with recurrent pericarditis [median age 32 years, interquartile range (IQR) 18.5; 67.6% females, all Caucasian, idiopathic aetiology in 71.1%] with a median number of recurrences of 5 (IQR 2). Overall, 16 patients (14.8%) had variants in genes related to the inflammatory response. Eleven variants were located in genes already associated with recurrent pericarditis (*NLRP3*, *TNFRSF1A* and *MEFV*) and five in inflammation/immunodeficiency-related genes (*IFIH1, NFKBIA, JAK1, NOD2* and *ALPK1*). Furthermore, we identified 10 patients with variants located in genes associated with conduction system-related diseases, and 22 variants in 21 patients with genes associated with heart structural-related diseases.

**Conclusion:**

In this first observational study using WES to assess genetic variants in patients with multiple recurrences of pericarditis, about 15% of patients bore at least one variant that may be related to the disease. These findings highlight the importance of addressing the role of genetic predisposition in recurrent pericarditis. Moreover, 28.7% of patients carry variants in different cardiac genes, worthy of a deeper investigation.

## Graphical abstract

**Figure:**
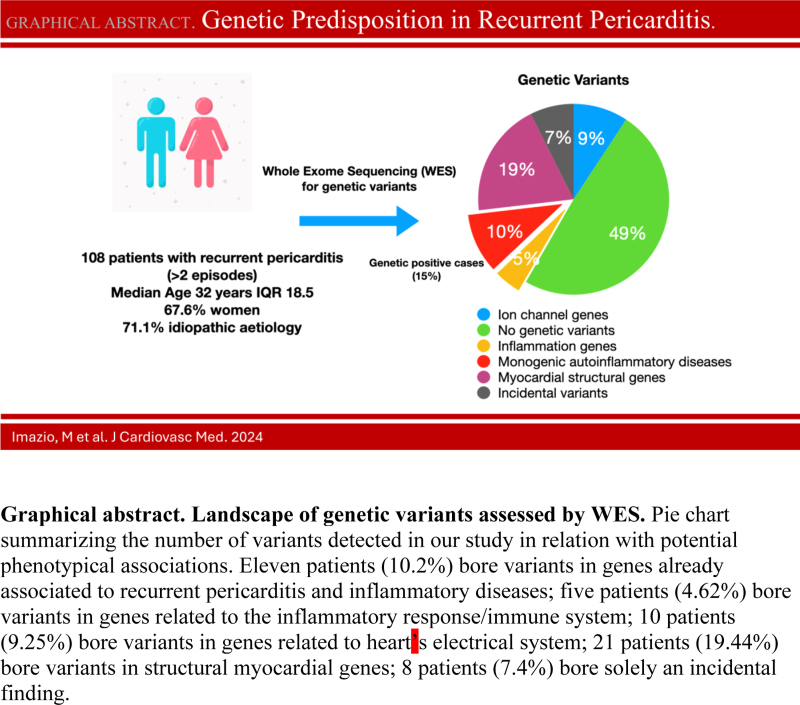

## Introduction

Recurrent pericarditis is one of the most troublesome complications of pericardial syndromes. It develops in 15–30% of patients after a first episode of acute pericarditis,^1,2^ and its frequency is estimated to be 25–50% of cases with recurrent pericarditis.^3,4^ Notably, approximately 6% of patients with recurrent disease will develop multiple recurrences (at least three) and a prolonged course of the disease for >1–2 years.^5^ It could be considered a rare cardiovascular disease since its estimated incidence is about 5–6 cases/100 000 inhabitants.^2–4,6–8^

Autoinflammatory and autoimmune mechanisms play a role in cases with multiple recurrences,^9–11^ especially in patients with corticosteroid-dependent and colchicine-resistant cases^12–14^ where anti-IL1 agents, such as anakinra and rilonacept, have demonstrated a high efficacy.^15,16^

However, a possible genetic predisposition seems implicated in such cases with multiple recurrences, since a family history of recurrent pericarditis has been reported in up to 10% of cases.^17,18^ Currently, genetic variants have been evaluated in a limited subset of genes, able to explain only about 5% of cases. These variants are clustered in genes related to autoinflammatory diseases, such as *TNFRSF1A*,^18^*MVK*, *NLRP3*,^19^ and the Interleukin 1 Gene Locus.^20^ Despite some progress in recent years, the current knowledge on the putative genetic basis of this disease is far from being elucidated.

Given these premises, the aim of the present study is to describe the genetic landscape of patients with idiopathic recurrent pericarditis and at least two recurrences. A three-tier analytical strategy will be performed assessing variants from: genes already associated with this disease, a subset of genes related to inflammation and autoimmunity and whole exome sequencing (WES).

## Materials and methods

### Study population and design

This is an observational cohort study including consecutive adult patients (>18 years) with at least two episodes of recurrent pericarditis. All patients were enrolled at the Cardiothoracic Department, University Hospital Santa Maria della Misericordia (Udine, Italy) from October 2022 to August 2023 and sent for genetic testing at the Institute of Medical Genetics, University Hospital Santa Maria della Misericordia (Udine, Italy). According to the 2015 European Society of Cardiology (ESC) guidelines for the diagnosis and treatment of pericardial diseases, recurrent pericarditis was diagnosed with a documented first episode of acute pericarditis, a symptom-free interval of 4–6 weeks or longer and evidence of subsequent recurrence despite guideline-based medical treatment. Family and personal history was collected with an emphasis on concomitant autoimmune diseases and pericarditis history (including aetiology, clinical features, number of recurrences, management, and outcomes). Informed consent to molecular analysis according to the Helsinki Declaration was obtained from all patients. The study was approved by the Institutional Review Board of the Department of Medicine at the University of Udine (Prot. IRB 200/2023).

### DNA extraction and next-generation sequencing

Genomic DNA was extracted from peripheral blood samples collected into 10 ml K2 Ethylenediaminetetraacetic acid collection tubes using the QIAsymphony SP/AS instrument (Qiagen, Hilden, Germany) according to the manufacturer's instruction. DNA concentration was estimated using the Qubit dsDNA HS Assay Kit on a Qubit 4.0 Fluorometer (Thermo Fisher Scientific, Waltham, MA, USA). WES was performed in all cases. A detailed description of the methodology of genetic analysis is reported as supplementary web addenda. All samples underwent WES. Variants were confirmed with orthogonal methods (Sanger sequencing and qPCR).

### Statistical analysis

Continuous variables were expressed as mean ± standard deviation (SD) or median and interquartile range (IQR) according to data distribution. The data were analysed using the Shapiro–Wilk test to verify the normal distribution. Categorical variables were presented as absolute numbers and percentages. The Student *t*-test or the Mann–Whitney *U* test was used to compare continuous variables between groups, as appropriate. Comparison of categorical variables was performed by chi-squared analysis or the Fisher exact test, as appropriate.

Analyses were performed using Stata 18.0 (Stata Corp LP, College Station, TX, USA) and MedCalc Statistical Software version 22.020 (MedCalc Software Ltd, Ostend, Belgium; https://www.medcalc.org; 2024).

## Results

### Study population

Overall, 108 patients diagnosed with recurrent pericarditis were included in this study. The baseline clinical features of the studied population are reported in Table 1. Patients had a median age of 32 (IQR 18.5) years, 67.6% (73/108) were females and all were Caucasian. The reported aetiology of pericarditis was idiopathic in 74 patients (71.1%), postvaccination in 16 patients (14.8%), and postcardiac injury syndrome in 15 cases (13.9%). An inflammatory phenotype was detected in 85 cases (78.7%) with elevation of C-reactive protein. At the time of genetic assessment, medical therapy included: nonsteroidal anti-inflammatory drugs (NSAIDs) in 103 (95.4%), colchicine in 104 patients (96.3%), corticosteroid therapy in 89 cases (82.4%), anakinra in 53 cases (49.1%), and iv immunoglobulins in 7 cases (6.5%). Being complicated cases of pericarditis, more than one drug class was prescribed in a single patient. The median number of recurrences was five (IQR 2) with a median duration of the disease of 4.9 years.

**Table 1:** Baseline features of patients enrolled in this cohort (*n* = 108)

Sex, *n* (%)	
Female	73 (67.6%)
Male	35 (32.4%)
Age at onset, median (IQR)	32 (18.5)
Caucasian, *n* (%)	108 (100.0%)
Family history of pericarditis, *n* (%)^a^	5 (4.6%)
Comorbidities, *n* (%)^b^	65 (60.2%)
Aetiology, *n* (%)	
Idiopathic	77 (71.3%)
Post vaccination	16 (14.8%)
Postcardiac injury syndrome (PCIS)	15 (13.9%)
Inflammatory phenotype with elevated CRP, *n* (%)	85 (78.7%)
Chest pain, *n* (%)	108 (100.0%)
Pericardial effusion, *n* (%)	64 (59.2%)
Polyserositis, n(%)	28 (25.9%)
Fever, *n* (%)	59 (54.6%)
First episode management, *n* (%)	
NSAID	100 (92.6%)
Colchicine	103 (95.4%)
Corticosteroids	7 (6.5%)

CRP, C-reactive protein; NSAIDs, nonsteroidal anti-inflammatory drugs.

a First-degree relative with a confirmed episode of acute pericarditis.

b See online table.

Previous corticosteroid dependence, defined as inability to taper or withdraw the drug without a relapse, was recorded in 46 patients (42.6%). A previous complicated course of pericarditis was recorded in nine cases (8.3%). Additional associated conditions are reported in online Table 1.

### Genetic studies

To identify the possible genetic bases of recurrent pericarditis, WES was performed.^21^ A three-layer analytical approach was considered. In a first analytical setting, variants in genes already associated with recurrent pericarditis were analysed. Eleven variants in *NLRP3*, *TNFRSF1A* and *MEFV* genes were assessed in 11 different patients (10.2%). These genes are known to be respectively associated with the cryopyrin-associated periodic fever syndrome (CAPS), the TNF Receptor-Associated Periodic Syndrome (TRAPS) and the Familial Mediterranean fever (FMF). Three likely pathogenic (LP) variants were assessed in *MEFV* (*n* = 1) and *NLRP3* (*n* = 2) genes. Moreover, a duplication involving the complete *MEFV* coding region was assessed and classified as a variant of unknown significance (VUS).

Besides, the same *TNFRSF1A* variant (NM_001065.4:c.362G>A) was assessed in seven patients. While pathogenic (P) and LP variants altering cysteine residues are associated with more severe phenotypes, variants of unknown significance or low-penetrance variants may play a broader role in inflammation. This variant is a well known low-penetrance mutation with an estimated frequency in the general European (non-Finnish) population of 1.8% (GnomAD). Notwithstanding, it has been detected with a 4.33 times higher frequency in our cohort (6.5%; *P* = 0.0003), suggesting a possible involvement in the pro-inflammatory status predisposing for recurrent pericarditis.

Thus, 10 out of 11 variants were already reported in the literature,^22–25^ with the exception of the frameshift variant in *NLRP3* (NM_004895.5:c.1953delC). All these data are summarized in Table 2.

**Table 2:** Genetic alterations in genes already associated with recurrent pericarditis

ID	Variant	Zigosity	ACMG classification
G22-2523	*MEFV* (NM_000243.3):c.1016C>T p.(Ser339Phe)^17^	0.5	LP (PS4 PM2)
G23-1159	NC_000016.10:g.3191321_3300615dup^a^^18^	0.5	VUS
G22-2672	*NLRP3* (NM_004895.5): c.1942G>T p.(Asp648Tyr)^19^	0.5	LP (PM1 PP2 PM2 PP3)
G22-2803	*NLRP3* (NM_004895.5):c.1953delC p.(Lys652ArgfsTer70)	0.5	LP (PVS1 PM2)
G22-2599	*TNFRSF1A* (NM_001065.4):c.362G>A p.(Arg121Gln)^20^	0.5	B (PM5 BA1 BS2 BP6)
G23-0385	*TNFRSF1A* (NM_001065.4):c.362G>A p.(Arg121Gln)^20^	0.5	B (PM5 BA1 BS2 BP6)
G23-0753	*TNFRSF1A* (NM_001065.4):c.362G>A p.(Arg121Gln)^20^	0.5	B (PM5 BA1 BS2 BP6)
G23-0732	*TNFRSF1A* (NM_001065.4):c.362G>A p.(Arg121Gln)^20^	1	B (PM5 BA1 BS2 BP6)
G23-1146	*TNFRSF1A* (NM_001065.4):c.362G>A p.(Arg121Gln)^20^	0.5	B (PM5 BA1 BS2 BP6)
G23-2325	*TNFRSF1A* (NM_001065.4):c.362G>A p.(Arg121Gln)^20^	0.5	B (PM5 BA1 BS2 BP6)
G23-3315	*TNFRSF1A* (NM_001065.4):c.362G>A p.(Arg121Gln)^20^	0.5	B (PM5 BA1 BS2 BP6)

a Including the complete coding region of *MEFV.*

Subsequently, in the second analytical setting, in all patients, variants in inflammation and immunodeficiency associated genes were analysed. Two LP variants in *IFIH1* and *ALPK1* and three VUS in *NFKBIA*, *JAK1* and *NOD2* genes were detected (Table 3). All variants are extremely rare in GnomAD (<0.01%), and none of them has been reported in the scientific literature yet.

**Table 3:** Variants in genes associated with other inflammatory/immunological conditions diseases

ID	Variant	Zigosity	ACMG classification
G23-520	*IFIH1* (NM_022168.4):c.1271del p.(Leu424TrpfsTer35)	0.5	LP (PVS1 PM2)
G23-1143	NC_000014.9:g.35307994_35405593dup^a^	0.5	VUS
G23-1495	*JAK1* (NM_002227.4):c.242A>G (p.Tyr81Cys)	0.5	VUS (PM2 PP2)
G23-1507	*NOD2* (NM_001370466.1):c.947T>C p.(Leu316Pro)	0.5	VUS (PM2)
23G1895	*ALPK1* (NM_025144.4):c.1948_1949dupCT p.(Gln651PhefsTer22)	0.5	LP (PVS1 PM2)

a Including the complete coding region of *NFKBIA* gene.

Finally, the complete set of variants identified by WES was analysed for all patients. Unexpectedly, we found that about 29% of samples harboured a variant in genes associated with different cardiac diseases, that is, cardiomyopathies and channelopathies.

Overall, we detected 10 variants in genes related to the heart conduction system. Five of them are classified as P or LP according to ACMG guidelines and are located in *SCN5A*, *KCND2*, *CALM2*, *TRPM4*, and *KCNQ1* genes. Variants in these genes are related to Brugada syndrome, long QT syndrome 15, progressive familial heart block type IB and long QT syndrome 1.^26–28^ Moreover, five are variants of unknown significance (VUS) and were assessed in *KCN5A*, *TRPM4* and *SCNA5* genes.

Furthermore, 22 variants in genes related to heart structural diseases were found in 21 patients. Twelve out of 22 are classified as deleterious by ACMG and are located in *DSC2*, *LMNA*, *TNNT2*, *ACTA2*, *BRAF*, *MIB1*, *RYR1*, *FBN1* and *TTN* genes. Variants in these genes are related to right ventricle arrhythmogenic cardiomyopathy, dilated cardiomyopathy, smooth muscle dysfunction syndrome, Noonan syndrome, left ventricular noncompaction, congenital myopathy, and Marfan syndrome. Instead, 10 variants were predicted to be VUS and are located in *JUP*, *DES*, *CTBP2*, *DSP*, *FBN1*, *MYBPC3*, *GATA5*, *MIB1* and *TTN* genes.^29–35^ One patient harboured two different variants: a deleterious variant in *TTN* gene and a VUS in *MYBPC3* gene (online-only Table 2). Besides variants putatively related to patient phenotype, incidental findings were highlighted in 10.2% of patients (*n* = 11/108), e.g. genetic variants unrelated to pericarditis, but of potential medical relevance. These heterozygous variants are located in genes causing dominant conditions, such as *LTBP3*, *CHD5*, *SCN1A*, *TBC1D8B*, *COL7A1*, *CLCN1*, *COL1A1*, *COL9A3*, *SDHA*, *EBP* and *ITPR3* genes^36–40^ (online-only Table 3).

Overall, five variants in genes already associated with recurrent pericarditis were assessed in 10.2% patients (*n* = 11/108) together with five variants in genes related to inflammation/immunodeficiency disorders in 4.6% of patients (*n* = 5/108). Overall, about 15% of patients bore a genetic variant (*n* = 16/108) that could be related to the assessed phenotype (multiple recurrences of pericarditis). No specific clinical feature was associated with these variants (supplemental material table). Moreover, 32 additional variants, 16 of which are predicted to be deleterious, were highlighted in in 31 patients (28.7%), mostly related to hereditary cardiac disorders and worthy of a deeper investigation. These findings are summarized in the graphical abstract.

## Discussion

Recurrent pericarditis is the most troublesome complication of pericarditis, affecting 15–30% of patients with a first episode of acute pericarditis and up to 50% of those with recurrences. A family history of pericarditis has been reported in up to 10% of cases raising suspicion of a possible genetic cause. To date, targeted analytical approaches have been published focusing on specific gene panels to evaluate the presence of monogenic autoinflammatory conditions, such as FMF, and TRAPS, that could manifest with recurrent pericarditis. In fact, these monogenic autoinflammatory conditions are able to explain 5–10% of patients with recurrent pericarditis. Nevertheless, the genetic basis of this disease is far from been elucidated.

In this scenario, omics technologies such as WES and WGS sequencing are preferred in those conditions in which the boundaries between research and diagnostics are faint, for example, analysing recurrent pericarditis-affected families. Indeed, WES is nowadays considered the first-tier analysis for hereditary disorders.^41^ But, although, on the one hand, WES allows us to increase the diagnostic rate of these conditions, on the other hand, it is associated with a discrete number of VUS and incidental findings.^42^ These are indeed an important challenge for both geneticists and cardiologists, who are required to periodically reassess the pathogenicity of these variants. Notwithstanding, omics technologies are deemed to be the cornerstone of genetic discovery, allowing knowledge on the pathogenesis of more complicated diseases to improve. All this enables a targeted and more successful medical therapy for pericarditis-burdened patients and their relatives.

In the last decade, knowledge of the genetic basis of cardiac inflammation has improved. In fact, myocarditis due to mutations in genes encoding desmosomes has recently been reported in the context of arrhythmogenic cardiomyopathies. In this view, it is interesting to highlight the role of e.g. *DSP*, *DSG2* and *PKP2* mutations in acute (recurrent) myocarditis. Moreover, genes already associated with cardiomyopathies (*BAG3*, *DSP*, *PKP2*, *RYR2*, *SCN5A*, or *TNNI3*) have been reported as mimicking acute myocarditis.^43–45^ Surprisingly in our series, we found a large number of patients carrying variants predicted to cause electrical or structural cardiac anomalies. Although this study cannot provide any evidence on the relationship between the conduction system and structural myocardial gene variants and pericarditis, the finding deserves further investigations and replication analysis.

To our knowledge, this observational study is the first evaluating patients affected by recurrent pericarditis by WES. In this study, we observed genetic variants in genes related to inflammatory/ immunological conditions in about 15% of patients with multiple recurrences of pericarditis: 11 of them had genetic variants already associated with recurrent pericarditis, and novel putative genes were found in 5 cases. Interestingly a single variant in the *TNFRSF1A* gene (NM_001065.4:c.362G>A) has been found in seven patients of our series (*P* = 0.0003), suggesting that it might be a possible predisposing factor for recurrent pericarditis.

Moreover, we detected 10 variants in genes related to the heart conduction system, and 22 variants affecting heart structural genes. These observations confirm that patients with multiple recurrences of pericarditis have a predisposing genetic background, which needs to be better clarified in additional focused research. Genetic testing should be especially considered for patients with multiple recurrences, young age at the onset, and inflammatory phenotype of presentation. Assessment of genetic variants may be helpful to individualize and target medical therapy of pericarditis (e.g. prolonged anti-inflammatory therapy targeted at the inflammasome in LP/P variants related to autoinflammatory diseases) in specific cases.^46,47^

## Limitations

This study has potential limitations. First, it has a single centre, retrospective design with a limited sample size. Patients with multiple recurrences of pericarditis were referred to our tertiary centre from all the country, so we included more complicated and severe cases. Nevertheless, this study highlights that a strict selection of these cases might improve the detection of rare potential causative variants. Of course, additional data are required to better characterize the involvement of these genetic variants in the pathogenesis of recurrent pericarditis.

## Conclusion

In conclusion, this is the first observational study using WES to assess variants in a cohort of patients affected by multiple recurrences of pericarditis. In this setting, about 15% of patients harboured a genetic variant in genes associated with inflammatory/immunological conditions, possibly explaining the phenotype, but an additional 28.7% of patients displayed a genetic alteration in cardiac-related genes that might have a role in the pathogenesis of the disease, highlighting the importance of additional studies to address the role of genetic predisposition in patients with multiple recurrences of pericarditis.

### Conflicts of interest

There are no conflicts of interest.

## Supplementary Material

Supplemental Digital Content

## Supplementary Material

Supplemental Digital Content
