# Supplementary material for: Genetic variants in patients with recurrent pericarditis
Source: J Cardiovasc Med (Hagerstown). 2024 Sep 17;25(11):799–804. doi: 10.2459/JCM.0000000000001669 (PMC11581433; doi:10.2459/JCM.0000000000001669)
Supplement: Supplemental Digital Content [file jcarm-25-799-s001.docx]

WEB-ONLY SUPPLEMENTAL MATERIALS

GENETIC ANALYSIS DESCRIPTION AND TABLE OF COMPARISON OF CLINICAL FEATURES IN PATIENTS WITH OR WITHOUT LP/P VARIANTS RELATED TO PERICARDITIS

We used a Whole Exome Sequencing approach and we confirmed the detected variants with the gold standard technique (Sanger sequencing and qPCR).

For Whole Exome Sequencing and data analysis, barcoded libraries were generated from 50 ng of DNA per sample. The exonic regions and flanking splice junctions (±25bp flanking each exon) of about 22’000 coding genes were captured using the WholEX pro sequencing kit (4bases SA, Manno, Switzerland). Sequencing was performed in paired-end 2 × 150 bp on a NextSeq system (Illumina Inc, San Diego, CA, USA). Reads were aligned to human genome build GRCh38/hg38 and variant calling were performed with the Varsome Clinical platform (Saphetor SA, Lausanne, Switzerland). Variant annotation and classification were performed with eVai (enGenome, Pavia, Italy). A minimum depth coverage of 20X, a minimum alternate allele frequency of 20% (VAF≥ 20%) and variants with frequency < 3% in population-based databases (i.e., gnomAD) were retained for further evaluation. I*n silico* gene panels were extracted from PanelApp (<https://panelapp.genomicsengland.co.uk/>). Were selected only heterozygous variants in dominant, homozygous / compound heterozygous in recessive and hemizygous in X-linked genes. The following public databases were used for the interpretation of the variants: HGMD Professional (https://my.qiagendigitalinsights.com/bbp), LOVD (<https://databases.lovd.nl/shared/genes>) and ClinVar (https://www.ncbi.nlm.nih.gov/clinvar/). Variants were classified according to the American College of Medical Genetics and Genomics (ACMG) guidelines^21^ using Varsome premium (<https://varsome.com/>) and Franklin by genoox (https://franklin.genoox.com/clinical-db/home) webtools.

**Sanger sequencing.** Amplification was performed using 50 ng of DNA and GoTaq® Colorless Master Mix (Promega, Madison, WI, USA). PCR primer sequences are available on demand. The amplified products were analysed by direct sequencing using the Big Dye Terminator Cycle Sequencing Kit v3.1 and capillary electrophoresis on the 3,500 Dx Series Genetic Analyzer (Applied Biosystems, Waltham, MA, USA).

**Quantitative Polymerase Chain Reaction (qPCR).** Amplification was performed using 10 ng of DNA and PowerUp SYBR Master Mix (Thermo Fisher Scientific, Waltham, MA, USA). PCR primer sequences are available on demand. Samples and controls were run in triplicate. Dissociation curves were generated to ensure primer specificity. Amplification was performed on a QuantStudio 3 PCR system (Thermo Fisher Scientific, Waltham, MA, USA).  Copy number was calculated using the automated ΔΔCt method using the QuantStudio Design & Analysis Software.
For genes involved in inflammation / immunodeficiency we selected genes to screen using PanelApp, while for other analysis' steps we applied an unbiased Whole Exome Sequencing.

E-table. Comparison of clinical features in patients with or without LP/P variants related to pericarditis.

| Feature | LP/P genetic variant related to pericarditis | | p |
| --- | --- | --- | --- |
|  | Yes (n=16) | No (n=92) |  |
| Age (mean ± SD) | 31.6±10.7 | 34.6±11.1 | 0.3059 |
| Female gender | 13 (81.3%) | 60 (65.2%) | 0.2081 |
| Family history of pericarditis | 5 (31.3%) | 37 (40.2%) | 0.4991 |
| Number of recurrences | 5.0±1.9 | 5.2±2.1 | 0.7455 |
| Idiopathic aetiology | 9 (56.3%) | 67 (72.8%) | 0.1620 |
| Inflammatory phenotype | 12 (75.0%) | 73 (79.3%) | 0.6964 |
| Pericardial effusion | 11 (68.8%) | 54 (58.7%) | 0.4504 |
| Polyserositis | 3 (18.9%) | 26 (28.3%) | 0.4303 |

LP/P= likely pathogenetic/pathogenetic; SD= standard deviation.
