# Supplementary material for: Genetic variants in patients with recurrent pericarditis
Source: J Cardiovasc Med (Hagerstown). 2024 Sep 17;25(11):799–804. doi: 10.2459/JCM.0000000000001669 (PMC11581433; doi:10.2459/JCM.0000000000001669)
Supplement: Supplemental Digital Content [file jcarm-25-799-s002.docx]

**Online-only tables.**

Online Table 1. Concomitant atopic or autoimmune diseases

|  | Overall population  (n=108) |
| --- | --- |
| Recurrent tonsillitis in childhood | 9 |
| Unexplained juvenile periodic fevers | 6 |
| Polyallergies | 8 |
| Retinal Detachment | 3 |
| Uveitis | 2 |
| Strabismus | 1 |
| Keratoconus | 1 |
| Atopic dermatitis | 4 |
| Psoriasis | 5 |
| Vitiligo | 1 |
| Systemic lupus erythematosus | 2 |
| Polymyalgia Rheumatica | 2 |
| Rheumatoid arthritis | 2 |
| Psoriatic Arthritis | 2 |
| Still’s disease | 2 |
| Graves-Basedow disease | 2 |
| Hashimoto thyroiditis | 4 |
| Migraine with aura | 4 |
| Epilepsy | 1 |
| Asthma | 6 |
| Acute appendicitis in children | 4 |
| Irritable Bowel Syndrome | 2 |
| Previous Malignancy | 4 |
| Endometriosis | 3 |
| Spontaneous Abortion  Recurrent Spontaneous Abortion | 4  2 |

Online-only table 2. Variants assessed analyzing the entire WES associated to the cardiovascular system.

| **ID** | **VARIANT** | **ZIGOSITY** | **ACMG CLASSIFICATION** | **SUBGROUP** |
| --- | --- | --- | --- | --- |
| **G22-2525** | *SCN5A* (NM_000335.5):c.1700T>A p.(Leu567Gln) | 0.5 | LP (PS3 PP5 PM1 PM2) | HEART’S ELECTRICAL SYSTEM |
| **G22-2672** | *CALM2* (NM_001743.6):c.355G>A p.(Asp119Asn) | 0.5 | LP (PM1 PM2 PP3) | HEART’S ELECTRICAL SYSTEM |
| **G22-2673** | *TRPM4* (NM_017636.4):c.797-2A>G p.? | 0.5 | P (PVS1 PP3 PM2) | HEART’S ELECTRICAL SYSTEM |
| **G22-3017** | *KCNA5* (NM_002234.4):c.707T>C p.(Val236Ala) | 0.5 | VUS (PM2) | HEART’S ELECTRICAL SYSTEM |
| **G23-323** | *TRPM4* (NM_017636.4):c.1928_1930dup p.(Leu643dup) | 0.5 | VUS (PM2 PM4) | HEART’S ELECTRICAL SYSTEM |
| **G23-384** | *KCND2* (NM_012281.3):c.1567C>T p.(Gln523Ter) | 0.5 | LP (PVS1 PM2) | HEART’S ELECTRICAL SYSTEM |
| **G23-470** | *SCN5A* (NM_000335.5):c.2381T>C p.(Met794Thr) | 0.5 | VUS (PP3 PM2 PP2) | HEART’S ELECTRICAL SYSTEM |
| **G23-666** | *SCN5A* (NM_000335.5):c.1820G>T p.(Gly607Val) | 0.5 | VUS (PP3 PM2 PP2) | HEART’S ELECTRICAL SYSTEM |
| **G23-1421** | *KCNQ1* (NM_000218.3):c.1748G>A p.(Arg583His) | 0.5 | P (PS4 PM1 PP2 PM2 PM5 PP3) | HEART’S ELECTRICAL SYSTEM |
| **23G2678** | *SCN5A* (NM_000335.5):c.3490G>A p.(Glu1164Lys) | 0.5 | VUS (PM3 PP3 PP2) | HEART’S ELECTRICAL SYSTEM |
| **G22-2524** | *JUP* (NM_002230.4):c.1391G>A p.(Ser464Asn) | 0.5 | VUS (PM2 BP4) | HEART’S STRUCTURAL SYSTEM |
| **G22-2749** | *DES* (NM_001927.4):c.1372-3dupC p.? | 0.5 | VUS (PM2 BP6) | HEART’S STRUCTURAL SYSTEM |
| **G22_2885** | *CTBP2* (NM_022802.3):c.470_492del p.(Ala157ValfsTer7) | 0.5 | VUS (PM2) | HEART’S STRUCTURAL SYSTEM |
| **G22-2944** | *DSC2* (NM_024422.6):c.2491C>T p.(Gln831Ter) | 0.5 | LP (PVS1 PM2) | HEART’S STRUCTURAL SYSTEM |
| **G22-2945** | *TTN* (NM_001267550.2): c.28924_28930delAGTGGGAinsTCAGCTTTGCTAGTTC p.(Gly9643_Thr9644delinsAlaLeuLeuValPro) | 0.5 | VUS (PM4 PM2) | HEART’S STRUCTURAL SYSTEM |
| **G22-3015** | *LMNA* (NM_170707.4):c.977C>T p.(Ser326Leu) | 0.5 | LP (PM1 PP2 PM2 PM5) | HEART’S STRUCTURAL SYSTEM |
| **G23-0156** | *RYR1* (NM_000540.3):c.131G>A p.(Arg44His) | 0.5 | LP (PP3 PM2 PM5 PP2) | HEART’S STRUCTURAL SYSTEM |
| **G23-0157** | *DSP* (NM_004415.4):c.3344C>T p.(Thr1115Ile) | 0.5 | VUS (PM2) | HEART’S STRUCTURAL SYSTEM |
| **G23-0239** | *BRAF* (NM_004333.6):c.20del p.(Gly7AlafsTer47) | 0.5 | LP (PVS1 PM2) | HEART’S STRUCTURAL SYSTEM |
| **G23-0386** | *FBN1* (NM_000138.5):c.1856C>A p.(Thr619Asn) | 0.5 | VUS (PM1 PM2 PP2 BP6) | HEART’S STRUCTURAL SYSTEM |
| **G23-0393** | *MIB1* (NM_020774.4):c.1966_1967del p.(Asn656CysfsTer19) | 0.5 | LP (PVS1 PM2) | HEART’S STRUCTURAL SYSTEM |
| **G23-0593** | *TTN* (NM_001267550.2): c.89017C>T p.( p.Arg29673Ter) | 0.5 | P (PS4 PP1 PVS1 PM2) | HEART’S STRUCTURAL SYSTEM |
|  | *MYBPC3* (NM_000256.3):c.3480C>G p.(Ile1160Met) | 0.5 | VUS (PM2 PP3) | HEART’S STRUCTURAL SYSTEM |
| **G23-0655** | *GATA5* (NM_080473.5):c.1175G>A p.(Cys392Tyr) | 0.5 | VUS (PM2 PP3) | HEART’S STRUCTURAL SYSTEM |
| **G23-0955** | *TTN* (NM_001267550.2):c.40356_40358del p.(Pro13454del) | 0.5 | VUS (PM2 PM4) | HEART’S STRUCTURAL SYSTEM |
| **G23-1264** | *FBN1* (NM_000138.5):c.7754T>C p.(Ile2585Thr) | 0.5 | LP (PM1 PM2 PP2 PS4) | HEART’S STRUCTURAL SYSTEM |
| **G23-1422** | *TNNT2* (NM_001001430.3):c.832C>T p.(Arg278Cys) | 0.5 | LP (PS4 PP1 PS3 PM5 PP2) | HEART’S STRUCTURAL SYSTEM |
| **G23-1617** | *FBN1* (NM_000138.5):c.2921G>A p.(Arg974His) | 0.5 | LP (PM1 PP2 PM2 PM5 PP3) | HEART’S STRUCTURAL SYSTEM |
| **G23-1989** | *RYR1* (NM_000540.3):c.5555G>C p.(Gly1852Ala) | 0.5 | LP (PP3 PM1 PM2) | HEART’S STRUCTURAL SYSTEM |
| **G23-2134** | *TTN* (NM_001267550.2):c.83831dup p.(Ser27945LysfsTer2) | 0.5 | LP (PVS1 PM2) | HEART’S STRUCTURAL SYSTEM |
| **G23-2679** | *MIB1* (NM_020774.4):c.769G>A p.(Asp257Asn) | 0.5 | VUS (PM2) | HEART’S STRUCTURAL SYSTEM |
| **G23-2680** | *ACTA2* (NM_001613.4):c.729G>T p.(Glu243Asp) | 0.5 | LP ( PP3 PM2 PM1 ) | HEART’S STRUCTURAL SYSTEM |

Online-only table 3. Incidental findings assessed analyzing the entire WES.

| **ID** | **VARIANT** | **ZIGOSITY** | **ACMG CLASSIFICATION** |
| --- | --- | --- | --- |
| **G22-1914** | *TBC1D8B* (NM_017752.3):c.1383G>A p.(Trp461Ter) | 0.5 | P (PVS1 PM2 PP5 BS2) |
| **G22-2524** | *CHD5* (NM_015557.3):c.4180C>T p.(Arg1394Ter) | 0.5 | LP (PVS1 PM2) |
| **G22-2802** | *COL7A1* (NM_000094.4):c.3403+2T>G p.? | 0.5 | LP (PVS1 PM2) |
| **G22-2944** | *SCN1A* (NM_001165963.4):c.2522C>T p.(Thr841Met) | 0.5 | LP (PM1 PP2 PM2 PM5 PP3 PP5 BP6) |
| **G22-3016** | *CLCN1* (NM_000083.3):c.1649C>T p.(Thr550Met) | 0.5 | P (PS4 PP1 PS3 PM1 PM2 PP2 PM5 PP3) |
| **G23-0086** | *COL1A1* (NM_000088.4): c.3040C>T p.(Arg1014Cys) | 0.5 | P (PS4 PP1 PS3 PM2 PP3 PP2) |
| **G23-0594** | *COL9A3* (NM_001853.4):c.346-1G>A p.? | 0.5 | LP (PVS1 PM2) |
| **G23-0667** | *SDHA* (NM_004168.4):c.457-2_457del p.? | 0.5 | P (PS4 PVS1 PM2) |
| **G23-0798** | *EBP* (NM_006579.3):c.298C>A p.(Leu100Ile) | 0.5 | LP ( PM1 PM5 PP3 PM2) |
| **G23-1116** | *ITPR3* (NM_002224.4):c.6547dup p.(Glu2183GlyfsTer130) | 0.5 | LP (PVS1 PM2) |
| **G23-1895** | *LTBP3* (NM_001130144.3):c.3118T>G p.(Cys1040Gly) | 0.5 | LP (PP3 PM2) |
